# Regular and Irregular Use and Reasons for Discontinuation of Solifenacin Therapy in Patients with Overactive Bladder Managed by Urologists

**DOI:** 10.3390/ph17010116

**Published:** 2024-01-16

**Authors:** Mateusz Małkowski, Agnieszka Almgren-Rachtan, Magdalena Olszanecka-Glinianowicz, Jerzy Chudek, Piotr Chłosta

**Affiliations:** 1 Adamed Pharma S.A., 05-152 Czosnow, Poland; malkowski.m@proton.me; 2 Department of Pharmacovigilance, Europharma Research & Science Centre Co. Ltd., 40-061 Katowice, Poland; 3 Health Promotion and Obesity Management Unit, Department of Pathophysiology, Medical Faculty in Katowice, Medical University of Silesia, 40-055 Katowice, Poland; magolsza@gmail.com; 4 Department of Internal Medicine and Oncological Chemotherapy, Medical Faculty in Katowice, Medical University of Silesia, 40-055 Katowice, Poland; 5 Department of Urology, Jagiellonian University Medical College, 31-008 Krakow, Poland; piotr.chlosta@uj.edu.pl

**Keywords:** overactive bladder, overactive bladder pharmacotherapy, real-life data, everyday clinical practice, solifenacin, storage symptoms, urinary urgency, urinary incontinence

## Abstract

Solifenacin, a selective muscarinic receptor antagonist, is one of the best-tolerated and most effective medicines that relieve storage symptoms in patients with an overactive bladder (OAB). However, the persistence of solifenacin in daily clinical practice remains far below that reported in clinical trials. This study aimed to analyze the adherence of patients to the therapy and the reasons for solifenacin discontinuation and non-regular use in OAB patients managed by urologists. Data concerning non-compliance and the discontinuation of solifenacin, along with the reasons, were collected during two consecutive visits for 64,049 OAB outpatients. Over the two visits, 81.6% of the patients continued therapy, and 88.6% were taking solifenacin regularly. An age ≥ 75 yrs., the male sex, a rural or small-city dwelling, and a prescription of ≥10 mg predicted therapy continuation. The female sex, a higher education, a short or long duration of an OAB, and a non-idiopathic OAB predicted regular use. The persistence of nycturia and urinary incontinence during therapy predicted both discontinuation and non-regular use. Dissatisfaction with therapy was the most frequent reason for discontinuation. In conclusion, an initial prescription of solifenacin at a low dose reduces the chance of OAB symptom improvement and results in more frequent discontinuation. A high rate of discontinuation related to dissatisfaction suggests unrealistic expectations for OAB patients and insufficient education by urologists.

## 1. Introduction

Epidemiological studies conducted in Western European countries and the US have estimated the prevalence of the symptoms of an overactive bladder (OAB) at about 16% (17.4% and 15.6% in European women and men aged above 40 years and 16.9% and 16.0% in US women and men aged above 18 years, respectively) [1,2]. In addition, the NOBLE study showed an increase in the incidence of urge incontinence with aging in women over 44 years old from 2% to 19% and in men over 64 years old from 0.3% to 8.9% [2]. Of note, as much as 40% of individuals with signs of an OAB never reported complaints to their doctor, and among the remaining 60%, only 27% were treated [1]. An OAB, especially urinary urgency, negatively affects health-related quality of life (HRQoL), increases the occurrence of anxiety and depression and the utilization of healthcare services [3], and decreases productivity at work [4].

Currently, the mainstays of the treatment of an OAB, when behavioral therapy fails, are drugs with anticholinergic activity that block muscarinic receptors, namely muscarinic receptor antagonists (MRAs) [5,6,7]. These drugs relax the bladder and increase its functional capacity but not its post-void residual volume, inhibit uncontrolled contractions of the detrusor, and even have a local analgesic effect [8].

Solifenacin is a competitive antagonist that is selective for the M3 receptor subtype and has a long elimination half-life, allowing a once-a-day prescription [9]. Regardless of its selectivity, its use is associated with the frequent, dose-dependent occurrence of adverse drug reactions (ADRs), mostly including a dry mouth (26%), constipation (12%), and blurred vision (5%) [10].

The adherence to solifenacin, as well as other MRAs, is much higher in clinical trials than in daily clinical practice. Administrative databases show that 62–86% of patients discontinued MRAs within 12 months, with some of them switching to another MRA [11,12,13]. Similarly, the discontinuation rates were high among long-term nursing home (LTNH) residents, with an OAB of 66.5% [14].

Several studies have tried to identify the factors associated with the low persistence of MRAs. A younger age, the male gender, a low MRA efficacy, ADRs, and the cost of treatment were found to be associated with the risk of discontinuation [13,15,16]. Among LTNH residents, the participants of a urinary toileting program were less likely to discontinue the use of MRAs [14].

The introduction of second-entry products lowered the cost of MRA therapy, which might at least potentially increase the persistence and affect the spectrum of the causes of discontinuation. Of note, the discontinuation of MRAs and the reasons for their irregular use have not been previously assessed in Eastern European countries.

Therefore, this study aimed to analyze the adherence to solifenacin therapy and the reasons for discontinuation and irregular use in a large cohort of outpatients with an OAB managed by urologists in the Polish population.

## 2. Results

### 2.1. Study Group Characteristics

A total of 64,049 patients (52.2% men) with a mean age of 63 ± 12 years were included in this analysis (Table 1). Older adults comprised 51.6% of the study group, including 14.7% in the subgroup aged 75 years or more. The share of people living in rural areas and with only primary education was lower, considering the demographic structure of Polish society. 

Neurological disorders and bladder obstruction as the cause of OAB were reported in 63.3% of patients. Neurological disorders were more frequent in women than in men (23.5% vs. 15.8%; *p* < 0.001), while bladder obstruction was more frequent in men than in women (70.4% vs. 14.7%; *p* < 0.001). During therapy, urge incontinence was reported by 37.6% of subjects. Most patients had had OAB symptoms for over a year (73.3%). Solifenacin was prescribed in a daily dose of 5 mg in 28.3% or 10 mg in 71.6% of those coming to the first visit. Larger doses—20 mg daily (not recommended by the Summary Product Characteristics)—were prescribed occasionally, 0.1% of the time.

Of the analyzed group, 97.6% came for a second visit after about 3 months.

### 2.2. Characteristics of Patients Discontinuing Therapy

Over two subsequent visits, 18.4% (N = 11,796) of patients discontinued solifenacin therapy. There were fewer older adults and individuals with higher education, idiopathic OAB, and those with short duration of urinary symptoms, but more large-city dwellers and patients with OAB attributed to bladder obstruction and persisting symptoms of OAB during therapy among those discontinuing therapy (Table 1).

### 2.3. Characteristics of Patients Taking the Drug Regularly

Based on the Morisky scale [17], 88.6% of 52,253 continuing therapy patients were taking solifenacin regularly. There were more women, large-city dwellers, individuals with higher education, OAB attributed to neurological disorders, short duration of urinary symptoms, and those prescribed with higher solifenacin dose (10 mg or 20 mg), but fewer with persisting symptoms of OAB during therapy (Table 1).

### 2.4. Factors Associated with the Continuation of Solifenacin Therapy

In univariate analysis (excluding participants prescribed with solifenacin in the daily dose of 20 mg), older age (≥75 yrs.), other than a large-city place of residence, short (<1 yr.) or long (>5 yrs.) duration of OAB symptoms, and a prescription of 10 mg of solifenacin had an increased chance of solifenacin therapy continuation. By contrast, female sex, younger age (<65 yrs.), other than vocational education, non-idiopathic causes of OAB, and persisting symptoms during therapy were the risk factors associated with therapy discontinuation (Table 2).

In a stepwise backward multiple regression model, OAB age ≥ 75 yrs. [OR = 1.96 (95%CI 1.779–1.180)], rural or small-city dwelling [OR = 1.84 (95%CI 1.676–2.031); OR = 1.61 (95%CI 1.490–1.741), respectively], and prescription of 10 mg of solifenacin [OR = 1.52 (95%CI 1.431–1.617)] predicted the continuation of therapy. By contrast, OAB coexisting with bladder obstruction [OR = 0.32 (95%CI 0.293–0.341)], only primary education [OR = 0.38 (95%CI 0.342–0.863)], the persistence of urinary urgency during therapy [OR = 0.43 (95%CI 0.384–0.473)], and female sex [OR = 0.46 (95%CI 0.433–0.496)] were the strongest predictors of discontinuation (Table 2). 

### 2.5. Factors Associated with the Regular Use of Solifenacin

In univariate analysis (excluding participants prescribed with solifenacin in the daily dose of 20 mg), short (<1 yr.) or long (>5 yrs.) duration of OAB symptoms, prescription of 10 mg of solifenacin, OAB in the course of neurological disorders, higher education (secondary or greater), female sex, and older age (≥65 yrs.) increased the chances of the regular use of solifenacin. By contrast, large-city residences, the persistence of nycturia, urinary frequency, and incontinence were associated with irregular solifenacin use (Table 2).

In a stepwise backward multiple regression model, the persistence of nycturia during therapy [OR = 0.55 (95%CI 0.502–0.611)] and urinary incontinence [OR = 0.77 (95%CI 0.700–0.844)] were the strongest predictors of irregular solifenacin use. By contrast, short or long duration of OAB [OR = 1.61 (95%CI 1.452–1.802); OR = 1.41 (95%CI 1.239–1.628), respectively], non-idiopathic causes of OAB [OR = 1.34 (95%CI 1.177–1.519) for neurological cause, and OR = 1.26 (95%CI 1.114–1.442) for bladder obstruction], female sex [OR = 1.31 (95%CI 1.161–1.457)], and higher education [OR = 1.28 (95%CI 1.174–1.394)] predicted the regular use of the drug (Table 2).

### 2.6. Reasons for Solifenacin Therapy Discontinuation

The reasons for the discontinuation of therapy were obtained in 7319 (62.0%). Of those who reported the reason, 35.9% of patients had problems filing prescriptions (probably related to the COVID-19 pandemic). The most frequent, when omitting factors beyond the control of the patient, was a lack of efficacy (22.4%). Interpreting the conscious decision not to file the prescription and the occurrence of ADRs (4.4%) as dissatisfaction, we may state that 58.9% of those discontinuing were not satisfied with therapy. In addition, 10.2% observed a remission of OAB symptoms (Table 3). It is worth noting that patients did not report high costs as the reason for therapy discontinuation.

### 2.7. Reasons for Non-Regular Solifenacin Use

Reasons for non-regular solifenacin use were provided by 5191 subjects (86.8%). Discomfort associated with solifenacin use was reported as the cause of irregular use for 25.7% of the population. In addition, 25.4% reported the high cost of therapy with solifenacin as the reason for irregular use. Furthermore, 10.6% reported irregular use related to a long waiting period for a control visit to obtain the prescription. The complete list of reasons is presented in Table 3.

## 3. Discussion

The results presented in this paper show that not only the discontinuation of solifenacin but also its irregular use is frequent in patients with OAB in Poland. During a short period of about 3 months, 18.4% of patients managed by urologists already discontinued therapy, which supports previous findings concerning the rapid decline in OAB medication in the first months of pharmacotherapy [18], with a median persistence shorter than 100 days [19]. It should be noted that our data are more optimistic than those for the Medicare cohort, where approximately half the patients did not refill MRA within 30 days of the end of the initial prescription [20]. Perhaps this difference is the effect of a selection of patients in our cohort with OAB who may have benefited from therapy with solifenacin performed by urologists.

Our study shows that being aged 75 years and over, male, a rural or small-city dweller, and having a prescription of 10 mg of solifenacin predicts the continuation of therapy. The coexistence of bladder obstruction or neurological diseases, primary education, and the persistence of urinary urgency during therapy were the strongest predictors of therapy discontinuation. Of note, solifenacin was prescribed in patients with OAB associated with neurological disorders, which is not recommended by the Summary Product Characteristics, especially in myasthenia gravis and autonomic neuropathy. Lower discontinuation rates among the oldest treated with MRA have already been described in the Medicare cohort without significant multimorbidity [20]. Higher rates of solifenacin discontinuation among females with OAB is a novel finding; however, it was previously reported in cardiology trials [21] and in therapy with statins [22]. The explanation for this remains unclear.

Urology outpatient clinics in Poland are mostly located in middle-sized and large cities. Consequently, access to services for rural and small-city residents is more difficult than for larger-city inhabitants. Therefore, this group of patients was initially more motivated to start and continue treatment.

The most clinically important predictor of solifenacin therapy persistence in our study, due to its potential modifiability, was the prescribed dose. We have demonstrated that the use of 10 mg solifenacin predicts the continuation of therapy, while the persistence of urinary urgency predicts its discontinuation. This suggests that, after the initiation of therapy with a 5 mg dose, it should be escalated (doubled) during the first weeks of treatment, if it is not effective, to prevent discontinuation caused by patient dissatisfaction related to unsatisfactory efficacy. It seems that dose escalation should be considered earlier than in the START trial, where it took place after 4 weeks [23], keeping in mind that a large percentage of patients do not refill MRA within 30 days of the end of the initial prescription [20]. Alternatively, a high initial dose of 10 mg daily may be prescribed, with an eventual reduction if ADRs appear. In a randomized study, adverse events—mostly dry mouth—developed in 17% of men with LUTS associated with BPH and OAB symptoms during 12 weeks of therapy with 10 mg of solifenacin and 0.2 mg of tamsulosin [24].

It is worth noting that ADRs were rare causes of the discontinuation of solifenacin therapy in our cohort. Only 4.4% of patients discontinued therapy for this reason. The occurrence of ADRs was higher, but the severity of symptoms resulting from the localization of muscarinic receptors in many organs and the limited selectivity of solifenacin was probably milder and less troublesome than that of OAB.

Satisfaction with MRA therapy is the most important factor affecting therapy persistence in daily clinical practice. It is driven by two domains, namely ADRs—mostly persistent gastrointestinal adverse events (negatively)—and expected improvement, which is associated with a positive satisfaction score and general impression [25]. In a national survey performed in the US, dissatisfaction with MRA effectiveness was a major reason for OAB treatment discontinuation [26]. Patient expectations related to the therapy were probably unrealistic, with the anticipation of a complete resolution of bothersome symptoms. This suggests that patient education performed by urologists in this aspect is far from what patients expect. The message that should be conveyed includes establishing OAB as a manageable, but not curable, medical condition and that solifenacin may only reduce the severity of symptoms. These important messages may make expectations more realistic and perhaps improve therapy persistence. An assessment of the subjective measures of urinary urgency performed in the VUNUS study showed that solifenacin improved an urgency perception score in only 42% of patients, which is more frequent than placebo (30%) [27]. The lack of improvement in more than half of the patients, shown by the VUNUS study, further explains why a large percentage of patients starting therapy may be dissatisfied and discontinue treatment.

The second important clinical issue is the irregular use of solifenacin in patients continuing therapy. In our cohort, 11.6% of those who did not discontinue were taking the drug irregularly. Our study found that female sex, higher education, short (less than one year) and long (>5 years) duration of OAB symptoms, and non-idiopathic causes of OAB predicted regular use of the drug. The persistence of nycturia and urinary incontinence during therapy were the strongest predictors of irregular solifenacin use. By contrast, women were more frequently taking the drug regularly. Higher education level is a well-known factor associated with adherence to MRA and other drugs. Previous studies have reported better compliance to antimuscarinic therapy among individuals with higher incomes [28], which is highly related to educational level. Better adherence among patients with a short duration of OAB symptoms may be explained by a shorter period of therapy duration. The decline in adherence (based on pill counts) over time was previously demonstrated in patients with urgent urinary incontinence during a 6-month period [29]. Furthermore, higher rates of patients with a long history of OAB symptoms who regularly use solifenacin are probably caused by the selection of patients that present long-term persistence with the therapy. The lack of data concerning the duration of therapy with solifenacin in our project precludes in-depth analysis and the confirmation of this hypothesis.

An interesting observation is the more frequent regular use of solifenacin among patients with non-idiopathic causes of OAB. However, the explanation of this finding is beyond the scope of our research.

Finally, we showed that the persistence of nycturia and urinary incontinence during therapy were the strongest predictors of non-regular solifenacin use. In our opinion, the persistence of these symptoms may be related in some cases to non-adherence and, in others, reflects the occurrence of ADRs (discomfort associated with solifenacin use was reported by 25.7% of irregular users) or the limited (unsatisfactory) effectiveness of the therapy. In addition, we have shown that the relatively high cost of therapy with solifenacin is declared as the reason for the non-regular use in one quarter (25.4%) of the population. In Poland, both the original medicine (Vesicare^®^, Astellas Pharma, Tokyo, Japan) and more than ten secondary-entry products of solifenacin are available in pharmacies, and some of them are partially reimbursed for patients with OAB. Still, the cost of therapy may be perceived as high by some older patients with severe multimorbidity treated with polytherapy.

Our study has some limitations, including the short period covered by the analysis (about 3 months) that precludes the analysis of long-term therapy persistence and the lack of reported urodynamic studies. However, contrary to data-registry-based studies, we included an analysis of regular solifenacin use and a large group of patient-related predictors. In addition, our project was performed during the COVID-19 pandemic, which explains patients’ independent lack of filing prescriptions as one of the reasons for solifenacin discontinuation.

In conclusion: (1) the initial prescription of solifenacin at a low dose reduces the chance for OAB symptom improvement and results in more frequent therapy discontinuation. (2) A high rate of solifenacin discontinuation related to dissatisfaction suggests unrealistic expectations for OAB patients and insufficient education by urologists. (3) ADRs are more often the reason for irregular use than the discontinuation of solifenacin therapy.

## 4. Patients and Methods

This large survey was carried out from 1 August 2019 to 31 March 2022 by 397 urologists and 52 in-training residents in a group of 64,049 adult outpatients (33,448 men and 30,601 women) with OAB who were prescribed solifenacin during the previous visit. Patient agreement to participate in the survey was the only additional inclusion criterion. Dementia and the inability to obtain answers to questions in the questionnaire were the only exclusion criteria. The survey did not meet the criteria of a medical experiment and, therefore, did not require Bioethical Committee approval. The study organizer (Europharma Research & Scientific Centre Co. Ltd., Katowice, Poland) processed anonymized patient data.

### 4.1. Survey Procedures

Investigators (urologists) were recruited among doctors who had effectively collaborated in previous projects. The survey was supported by a study questionnaire filled out by investigators during two subsequent visits based on an interview and medical history. Data from eligible patients who refused to participate were not collected.

Investigators filled in patients’ questionnaires, which included age, educational level, place of residence, clinical data (period since the diagnosis of OAB, symptoms: urinary urgency, urinary frequency, nycturia, and urge incontinence), use of solifenacin, and main factors that affected its choice, compliance (based on the 8-point Morisky scale), and eventual discontinuation with the declared reason. During a subsequent visit, OAB symptoms and compliance with solifenacin based on the 8-point Morisky scale and eventual discontinuation were reassessed.

### 4.2. Data Analysis

Non-compliance was scored based on the Morisky scale. A score of at least 4 was classified as non-compliance.

Patients were considered to be taking medication (solifenacin) regularly when, during both assessments, they did not discontinue the use of solifenacin and were classified as compliant according to the Morisky scale [17]. Non-compliance during any assessments allowed for patients to be classified as taking the medication irregularly. Those who discontinued therapy with solifenacin before Visits 1 or 2 were scored as discontinuing therapy.

### 4.3. Statistical Analysis

A total of 64,049 questionnaires of patients with OAB previously prescribed solifenacin, without missing epidemiological data, were completed by the investigators that were included in this analysis. Patient questionaries showing other MRA prescriptions before the first visit [N = 12,509] or with missing data [N = 2482] were not included. Statistical analysis was performed using the STATISTICA 11.0 PL software (Tibco Software Inc, Palo Albo, CA, USA). No data imputation was performed. Statistical significance was set at a *p*-value below 0.05. All tests were two-tailed. Nominal and ordinal data were expressed as percentages, while interval data were shown as mean value ± standard deviation (SD). The distribution of variables was evaluated by the Shapiro–Wilk test. For the comparison of interval data, the Student’s *t*-test was used. Categorical variables were compared using χ2 test and χ2 test for trend. Odds ratios (OR) were calculated for two models: the continuation of therapy with solifenacin and in the subgroup of continuing therapy—regular use of solifenacin. Initially, univariate ORs were calculated. In the second step, multivariable models were created, including all variables used in univariate models, applying stepwise backward regression models (N = 54,424 and N = 49,243 for final models after the exclusion of records with missing data and patients prescribed with 20 mg of solifenacin, respectively).

## 5. Conclusions

Initial prescription of solifenacin at a low dose reduces the chances of OAB symptom improvement and results in more frequent therapy discontinuation. A high rate of solifenacin discontinuation related to dissatisfaction suggests unrealistic expectations of patients with OAB and insufficient education delivered by urologists. ADRs are more often the reason for irregular use of solifenacin than the discontinuation of the therapy.

## Figures and Tables

**Table 1:** Characteristics of the study group of patients prescribed solifenacin, subgroups taking the medicine on a regular or irregular basis, and those discontinuing therapy.

	All Patients	Taking Solifenacin Regularly	Not Taking Solifenacin Regularly	Discontinuing Solifenacin Therapy
		A	B	C
	(N = 64.049)	(N = 46,271)	(N = 5.982)	(N = 11.796)
Men (N; %)	33,448; 52.2	24,465; 52.9	3296; 55.1 **	5687; 48.2 ***
Age (years)	63 ± 12	64 ± 12	63 ± 13 ***	63 ± 11 **
Age <65 years (N; %)	30,984; 48.4	22,324; 48.3	2989; 49.9	5671; 48.1
Age 65–74 years (N; %)	23,647; 36.9	16,763; 36.2	2085; 34.9	4799; 40.7
Age ≥75 years (N; %)	9418; 14.7	7184; 15.5	908; 15.2 *	1326; 11.2 ***
Education level:				
Primary (N; %)	3452; 5.5	2306; 5.1	349; 6.0	797; 6.9
Vocational (N; %)	12,833; 20.4	9486; 20.9	1396; 23.9	1951; 16.9
Secondary (N; %)	31,737; 50.6	22,495; 49.5	2930; 50.1	6312; 54.5
Higher (N; %)	14,779; 23.5	11,097; 24.5	1166; 20.0 ***	2516; 21.7 ***
No data (N)	1248	887	141	220
Place of residence:				
Rural (N; %)	9092; 14.4	6547; 14.3	1062; 18.0	1483; 12.7
Small city; <25 thou. PR (N; %)	13,693; 21.6	10,076; 22.1	1449; 24.5	2168; 18.6
Medium city; 25–100 thou. PR (N; %)	16,426; 26.0	12,032; 26.3	1515; 25.6	2879; 24.6
Big city; >100 thou. PR (N; %)	24,071; 38.0	17,028; 37.3	1888; 31.9 ***	5155; 44.1 ***
No data (N)	767	588	68	111
Reason for OAB:				
Neurological (N; %)	12,251; 19.4	9071; 19.9	920; 15.5 ***	2260; 19.7
Bladder obstruction (N; %)	27,648; 43.9	19,239; 42.1	2586; 43.6 *	5823; 50.9 ***
Idiopathic (N; %)	23,118; 36.7	17,317; 38.0	2429; 40.9 ***	3372; 29.4 ***
No data (N)	1032	644	47	341
Persistence of symptoms:				
Urinary urgency (N; %)	55,209; 86.2	39,048; 84.4	5076; 84.9	11,085; 94.0 ***
Urinary frequency (N; %)	42,276; 66.0	29,236; 63.2	4132; 69.1 ***	8908; 75.5 ***
Nycturia (N; %)	36,367; 56.8	24,772; 53.5	4127; 69.0 ***	7468; 63.3 ***
Urinary incontinence (N; %)	24,055; 37.6	16,260; 35.1	2287; 38.2 ***	5508; 46.7 ***
Duration of OAB symptoms:				
>5 years (N; %)	9087; 14.4	6765; 14.8	783; 13.2	1539; 13.4
1–5 years (N; %)	37,078; 58.9	25,914; 56.9	3944; 66.5	7220; 62.7
<1 year (N; %)	16,851; 26.7	12,903; 28.3	1204; 20.3 ***	2744; 23.9 ***
No data (N)	1033	689	51	293
Solifenacin dose:				
5 mg (N; %)	18,113; 28.3	12,237; 26.4	2132; 35.6	3744; 31.7
10 mg (N; %)	45,883; 71.6	34,007; 73.5	3824; 64.0	8052; 68.3
20 mg (N; %)	53; 0.1	27; 0.1	26; 0.4 ***	0 ***

Statistical significance: B vs. A; C vs. A. * *p* < 0.05; ** *p* < 0.01; *** *p* < 0.001.

**Table 2:** Factors associated with the regular use and continuation of solifenacin therapy. Univariable and multivariable analyses (stepwise backward regression models). Patients prescribed with a 20 mg daily dose of solifenacin were excluded from this analysis.

		Chance for Regular Use OR (95%CI)		Chance for Continuation OR (95%CI)	
		Univariate	Multivariate	Univariate	Multivariate
Sex	Men	Ref.	Ref.	Ref.	Ref.
	Women	1.10 (1.046–1.166) ***	1.31 (1.161–1.457) ***	0.83 (0.797–0.864) ***	0.46 (0.433–0.496) ***
Age	<65 years	Ref.	Ref.	Ref.	Ref.
	65–74 years	1.09 (1.026–1.156) *	-	0.89 (0.849–0.926) ***	1.08 (1.019–1.150) **
	≥75 years	1.06 (0.979–1.146)	-	1.37 (1.287–1.467) ***	1.96 (1.777–1.180) ***
Education level	Vocational	Ref.	Ref.	Ref.	Ref.
	Primary	0.97 (0.853–1.097)	-	0.59 (0.539–0.650) ***	0.38 (0.342–0.435) ***
	Secondary	1.14 (1.064–1.220) ***	-	0.73 (0.693–0.775) ***	0.81 (0.751–0.863) ***
	Higher	1.40 (1.290–1.521) ***	1.28 (1.174–1.394) ***	0.91 (0.850–0.968) **	-
Place of residence	Big city	Ref.	Ref.	Ref.	Ref.
	Rural	0.67 (0.62–0.729) ***	-	1.33 (1.251–1.423) ***	1.84 (1.676–2.031) ***
	Small city	0.76 (0.707–1.818) ***	-	1.41 (1.331–1.488) ***	1.61 (1.490–1.741) ***
	Medium city	0.87 (0.807–0.932) ***	-	1.26 (1.201–1.330) ***	1.18 (1.109–1.269) ***
Cause of OAB	Idiopathic	Ref.	Ref.	Ref.	Ref.
	Neurological	1.38 (1.270–1.491) ***	1.34 (1.177–1.519) ***	0.78 (0.737–0.830) ***	0.56 (0.514–0.603) ***
	Bladder obstruction	1.04 (0.983–1.106)	1.26 (1.114–1.422) ***	0.64 (0.614–0.675) ***	0.32 (0.293–0.341) ***
Persistence of urinary urgency	No	Ref.	Ref.	Ref.	Ref.
	Yes	0.97 (0.899–1.045)	1.22 (1.076–1.368) **	0.35 (0.320–0.375) ***	0.43 (0.384–0.473) ***
Persistence of urinary frequency	No	Ref.	Ref.	Ref.	Ref.
	Yes	0.77 (0.729–0.819) ***	0.90 (0.815–0.991) *	0.56 (0.531–0.582) ***	0.63 (0.588–0.675) ***
Persistence of nycturia	No	Ref.	Ref.	Ref.	Ref.
	Yes	0.52 (0.491–0.552) ***	0.55 (0.502–0.611) ***	0.67 (0.640–0.695) ***	0.84 (0.784–0.889) ***
Persistence of urine incontinence	No	Ref.	Ref.	Ref.	Ref.
	Yes	0.89 (0.838–0.938) ***	0.77 (0.700–0.844) ***	0.62 (0.594–0.645) ***	0.65 (0.616–0.694) ***
Duration of OAB symptoms	1–5 years	Ref.	Ref.	Ref.	Ref.
	<1 year	1.62 (1.515–1.735) ***	1.61 (1.452–1.802) ***	1.31 (1.248–0.376) ***	1.19 (1.115–1.269) ***
	>5 years	1.30 (1.202–1.415) ***	1.41 (1.239–1.628) ***	1.22 (1.150–0.300) ***	-
Solifenacin dose	Low (5 mg)	Ref.	Ref.	Ref.	Ref
	High (10 mg)	1.55 (1.464–1.640) ***	1.50 (1.361–1.642) ***	1.29 (1.237–0.350) ***	1.52 (1.431–1.617) ***

Ref.—reference group; statistical significance: * *p* < 0.05; ** *p* < 0.01; *** *p* < 0.001.

**Table 3:** Reasons for irregular dosage and treatment discontinuation among solifenacin-prescribed patients with overactive bladder (OAB).

**Patients Not Taking Solifenacin Regularly**	**[N = 5982]**
High cost of treatment (less use of the drug will save money) (N; %)	1317; 25.4
To decrease the discomfort associated with its administration (N; %)	1336; 25.7
Lack of efficacy (no improvement) (N; %)	416; 8.0
Conscious breaks in treatment for several days—drug holidays (N; %)	1756; 33.8
Forgetting to take medicine (N; %)	4367; 84.1
Shift work system (N; %)	242; 4.7
Frequent trips (N; %)	304; 5.9
Communication error (N; %)	531; 10.2
No medicine, long waiting time for an appointment to obtain a prescription (N; %)	550; 10.6
Other (N; %)	64; 1.2
More than one reason (N; %)	2623; 50.5
No reason provided (N)	791
**Patients Discontinuing Solifenacin Therapy**	**[N = 11,796]**
Lack of efficacy—no improvement (N; %)	1642; 22.4
A conscious decision of not filling the prescription (N; %)	2347; 32.1
Symptoms remission (N; %)	743; 10.2
Adverse drug reaction (N; %)	325; 4.4
Complicated drug administration (N; %)	344; 4.7
Patients’ independent lack of filing a prescription (N; %)	2626; 35.9
The decision of another physician (N; %)	256; 3.5
Communication error concerning the continuous use of the drug (N; %)	958; 13.1
More than one reason (N; %)	576; 7.9
No reason provided (N)	4477

## Data Availability

The datasets are available from the Europharma Research and Science Centre Co. Ltd. on a reasonable request (agnieszka@europharma.edu.pl).
